# High Flow Nasal Cannula Decreased Pulmonary Complications in Neurologically Critically Ill Patients

**DOI:** 10.3389/fnhum.2021.801918

**Published:** 2022-01-04

**Authors:** Shuanglin Wang, Jingjing Yang, Yanli Xu, Huayun Yin, Bing Yang, Yingying Zhao, Zheng Zachory Wei, Peng Zhang

**Affiliations:** ^1^Department of Thoracic and Cardiovascular Surgery, Tianjin Medical University General Hospital, Tianjin, China; ^2^Department of Critical Care Medicine, Tianjin Medical University General Hospital Airport Hospital, Tianjin, China; ^3^Department of Cell Biology, College of Basic Medical Sciences, Tianjin Medical University, Tianjin, China; ^4^Department of Neurology, Affiliated Beijing Friendship Hospital, Capital Medical University, Beijing, China

**Keywords:** high-flow nasal cannula therapy, neurological critical ill, pulmonary complication, hypoxemia, neurological function

## Abstract

**Objective:** Pulmonary complications could badly affect the recovery of neurological function and neurological prognosis of neurological critically ill patients. This study evaluated the effect of high-flow nasal cannula (HFNC) therapy on decreasing pulmonary complications in neurologically critically ill patients.

**Patients and Methods:** The patients admitted to the intensive care unit (ICU) with serious neurological disease and receiving oxygen therapy were retrospectively reviewed (Ethical No. IRB2021-YX-001). Patients were divided into the HFNC group and the conventional oxygen therapy (COT) group. We analyzed the data within these two groups, including patients’ baseline data, short-term outcomes of respiratory complications, general outcomes including hospital stay, ICU stay and mortality, and neurological functions. To analyze the relevant factors, we performed multivariable logistic regression analysis.

**Results:** A total of 283 patients met the criteria, including 164 cases in the HFNC group and 119 cases in the COT group. The HFNC group had remarkably less mechanical ventilation requirement with lower phlegm viscosity. Even more, ICU stay and total hospital stay were significantly shortened in the HNFC group.

**Conclusion:** HFNC decreased pulmonary complications in neurologically critically ill patients and improved recovery of neurological function and neurological prognosis.

## Introduction

The neurologically critically ill patient is more susceptible to pulmonary complications, such as hypostatic pneumonia, acute respiratory distress syndrome (ARDS), aspiration pneumonia and pulmonary atelectasis (Stueber et al., 2017). Pulmonary complications with a respiratory function disturbed may lead to hypoxemia and hypercapnia (Hu et al., 2017). Subsequently, an oxygen-poor condition may also hamper the recovery and prognosis of neurological function in neurologically critically ill patients (Gaddam et al., 2015). Massive systemic inflammation factor release combined with neurologically critical conditions result in neurogenic pulmonary edema (an increase in pulmonary interstitial and alveolar fluid) of the patients. We try to explore an efficient way to reduce pulmonary complications and improve respiratory function, benefiting patients with neurologically critical conditions.

High-flow nasal cannula (HFNC) therapy involved a new heated and humidified system consisting of an air-oxygen blender, and a heated humidification equipment. Compared with Venturi-type masks, HFNC could provide a higher concentration of oxygen, more steady oxygen flow, better humidification function and reduce the workload of breathing (Chanques et al., 2013; Rittayamai et al., 2014). HFNC could provide precise and adjustable oxygen concentration to the patients while keeping positive airway pressure at a relatively low value (approximately 0.5∼1 cm H_2_O). HFNC could also improve patients’ functional residual capacity (FRC) (Maggiore et al., 2014).

In our clinical practice, HFNC seemed to be beneficial for keeping a humid airway and patient’s cough up sputum, as well as preventing the occurrence of hyperoxia and hypoxemia. Therefore, this study was conducted to investigate the role of HFNC in pulmonary complications in those neurologically critically ill patients.

## Patients and Methods

### Patients

The patients admitted to the ICU of Tianjin Medical University General Hospital Airport Hospital with serious neurological disease and who received oxygen therapy between May 2015 and December 2019 were retrospectively reviewed (Ethical No. IRB2021-YX-001). Patients were 17∼91 years old. The inclusion criteria included the patients admitted to ICU within the first 24 h after disease attack (include traumatic brain injury, spontaneous intracerebral hemorrhage, cerebral infarction, spontaneous subarachnoid hemorrhage, spontaneous subdural hematoma, and spontaneous epidural hematoma) who had hypoxemia and required oxygen therapy. The exclusion criteria were patients with trauma-induced respiratory failure who needed mechanical ventilation and were difficult to wean, patients with malignant tumors, and patients with a history of serious cardiopulmonary dysfunction. All signed informed consents were obtained.

The patients’ baseline data including age, gender, total hospital stay, ICU stay, APACHE II (Acute Physiology and Chronic Health Evaluation II) and oxygenation index, etc. were collected.

### Therapeutic Methods

According to the guidelines, the treatment of neurological damage included, proper control of intracranial pressure, reducing brain tissue edema, nutrition support, balancing of blood pressure, blood glucose, ion and *p*H, etc. The patients with traumatic brain injury and cerebral hemorrhage were treated with hemostatic medication. The patients with cerebral infarction were given anticoagulation therapy and antiplatelet therapy. For the patients with spontaneous subarachnoid hemorrhage, hemostatic medication and preventive treatment of cerebral vasospasm were adopted. Surgical operation was conducted if there were indications of operation in the patients with traumatic brain injury, cerebral hemorrhage, cerebral infarction or spontaneous subarachnoid hemorrhage. If any was diagnosed as intracranial aneurysm among the patients with spontaneous subarachnoid hemorrhage, aneurysm occlusion or interventional aneurysm embolization therapy was performed.

After the above treatment, the patients in the HFNC group received HFNC using Airvo and Optiflow Interface (Fisher & Paykel Healthcare Ltd., Auckland, NZ). The oxygen concentration and gas-flow rate were adjusted according to the level of PaO_2_ and SpO_2_ which were maintained at 85∼100 mm Hg (PaO_2_) and 95∼100% (SpO_2_). The gas temperature was set as 37°C. The patients in the conventional oxygen therapy (COT) group, received COT including oxygen inhalation through a nasal catheter and mask oxygen inspiration. The oxygen concentration was adjusted to keep PaO_2_ at 85∼100 mm Hg and SpO_2_ at 95∼100%. The oxygen therapy continued during the ICU stay.

### Follow-Up and Data Collection

The respiratory outcomes, including the incidence of pneumonia, mechanical ventilation, sputum viscosity was recorded. ΔGCS (changes in the Glasgow Coma Scale score at 14 days after the attack) was used as an indicator of short-term neurological outcome. The general outcomes were recorded including total hospital stay, ICU stay and in-hospital mortality.

### Statistical Analysis

Statistical analysis was performed using SPSS 25.0 (SPSS Inc., Chicago, IL, United States). Data were presented as mean ± SD for continuous variables in a normal distribution, as M (Q1∼Q3) for quantitative variables not obeying normal distribution frequencies, and as percentages for categorical variables. Independent *t*-test or Wilcoxon rank-sum test was used for comparison of continuous variables between the two groups. The chi-square test or Wilcoxon rank-sum test was used for the comparison of categorical variables between the two groups. Multivariable logistic regression analysis or multiple linear regression analysis was performed for outcome variables that were statistically significant to determine the influencing factors. *P*-value < 0.05 was considered statistically significant.

## Results

### Baseline Data of the Patients

**FIGURE 1:**
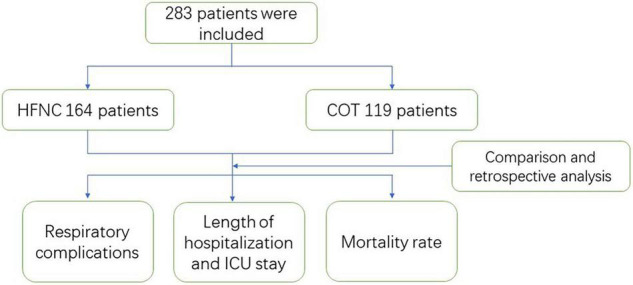
Study flow chart. HFNC, High flow nasal cannula; COT, Conventional oxygen therapy.

In this study, 283 patients met the inclusion criteria and were enrolled into the study, including 164 cases in the HFNC group and 119 cases in the COT group. APACHE-II, GCS admission, and oxygenation index showed significant differences within these two groups (Table 1 and Figure 1).

**TABLE 1:** Comparison of clinical characters between COT group and HFNC group.

Baseline characteristics		COT	HFNC	*p*-value
Age (years)		53.83 ± 15.14	56.97 ± 16.17	0.096
Gender (F/M)		38/81	53/111	>0.05
APACHE-II		8.25 ± 3.98	9.80 ± 4.32	<0.01*
GCS at admission		10.64 ± 1.63	10.25 ± 1.63	0.049*
P/F^#^		354.81 ± 22.58	344.37 ± 26.32	<0.01*
Diagnosis	TBI	26	35	0.841
	ICH	52	77	
	CI	21	31	
	SAH	7	7	
	SDH	4	7	
	EPH	9	9	

*<0.05 indicate the significant result in the assessment. ^#^PaO_2_/FiO_2_, Respiratory failure index.

### Outcome Measurement and Comparison of Between the Two Groups

#### Respiratory Outcomes

Compared with the COT group, the HFNC group had significantly less need for mechanical ventilation (*p* < 0.01) (Table 2), and lower phlegm viscosity (*Z* = 12.175, *p* < 0.01). But there was no statistically significant difference in the incidence of pneumonia during hospitalization between the two groups (*p* > 0.05) (Table 2).

**TABLE 2:** Outcome of respiratory complications, short term neurological prognosis, and general outcomes.

		COT	HFNC	*p*-value
Respiratory complications	Pneumonia	14/119	9/164	0.056
	Mechanical ventilation	10/119	2/164	<0.01*
ΔGCS		1.88 ± 1.30	2.34 ± 0.88	<0.01*
Length of hospitalization		15.50 ± 4.06	12.89 ± 3.80	<0.01*
Length of ICU stay		7.66 ± 3.47	5.81 ± 2.59	<0.01*
Mortality rate		2/119	1/164	>0.05

*<0.05 indicate the significant result in the assessment.

Our study primarily focused on the impact of HFNC on the respiratory system prognosis of patients with severe neurological diseases compared with traditional oxygen therapy. We conducted multivariable logistic regression analyses on the respiratory system prognosis (Tables 3–5), and found that: (1) Oxygen therapy was an important factor affecting the incidence of pneumonia during hospitalization (*p* = 0.009); (2) Oxygen therapy was an important factor affecting the occurrence of mechanical ventilation during hospitalization (*p* = 0.003); (3) Oxygen therapy was correlated with sputum viscosity as a critical parameter (correlation coefficient = −0.718, *p* < 0.01).

**TABLE 3:** Logistics regression analysis of mechanical ventilation.

	Intercept and variable	Coefficient	Odds ratio (95%CI)	*p*-value
Mechanical	Itercept	3.090	21.973	0.386
ventilation	Gender	0.995	2.705 (0.522–14.027)	0.236
	Age	0.030	1.030 (0.978–1.085)	0.262
	APACHE II	–0.038	0.963 (0.768–1.206)	0.740
	GCS_admission	–0.693	0.500 (0.269–0.930)	0.029*
	Diagnosis	–0.349	0.705 (0.348–1.429)	0.332
	Oxygen therapy	–2.416	0.089 (0.018–0.450)	0.003*

*<0.05 indicate the significant result in the assessment.

**TABLE 4:** Logistics regression analysis of pneumonia occurred during hospitalization.

	Intercept and variable	Coefficient	Odds ratio (95%CI)	*p*-value
Pneumonia	Itercept	–0.733	0.481	0.741
	Gender	0.282	1.326 (0.482–3.644)	0.584
	Age	0.007	1.007 (0.971–1.045)	0.699
	APACHE II	0.149	1.160 (1.012–1.331)	0.033*
	GCS_admission	–0.280	0.756 (0.523–1.092)	0.136
	Diagnosis	–0.250	0.779 (0.507–1.196)	0.253
	Oxygen therapy	–1.276	0.279 (0.107–0.728)	0.009*

*<0.05 indicate the significant result in the assessment.

**TABLE 5:** Logistics regression analysis of sputum viscosity.

	Intercept and variable	Coefficient	Odds ratio (95%CI)	*p*-value
Sputum	Itercept	0.185	−1.685	0.368
Viscosity	Gender	0.884	−0.124 (−0.761 to 0.514)	0.704
	Age	1.009	0.009 (−0.018 to 0.036)	0.515
	APACHE II	1.033	0.032 (−0.087 to 0.151)	0.595
	GCS_admission	0.692	−0.368 (−0.627 to −0.110)	0.005*
	Diagnosis	0.588	−0.531 (−2.130 to 1.068)	0.515
	Oxygen therapy	155.887	5.049 (4.006−6.092)	0.000*

*<0.05 indicate the significant result in the assessment.

#### Neurologic Outcome

For neurological function assessment, we used changes in GCS score at 14 days after onset as an indicator of short-term neurological outcomes, and we found that the HFNC group had better short-term neurological outcomes than the COT group (*p* < 0.01) (Table 2).

Developing a multivariable logistic regression model to analyze short-term neurological outcomes, we found that oxygen therapy was an important factor (*p* < 0.010) (Table 6), which indicates that oxygen therapy had an important effect on short-term neurological prognosis.

**TABLE 6:** Linear regression analysis on ΔGCS.

	Intercept and variable	B	SE	β	t	*p*-value
ΔGCS	Itercept	4.210	0.634		6.644	<0.010
	Gender	–0.270	0.137	–0.011	–0.195	0.846
	Age	0.018	0.005	0.254	3.260	< 0.010*
	APACHE II	–0.108	0.025	–0.417	–4.367	< 0.010*
	GCS_admission	–0.227	0.052	–0.338	–4.371	< 0.010*
	Diagnosis	0.020	0.047	0.025	0.417	0.677
	Oxygen therapy	0.485	0.127	0.219	3.807	< 0.010*

*<0.05 indicate the significant result in the assessment.

#### General Outcomes

The length of ICU stay was 7.66 ± 3.47 days in the COT group and 5.81 ± 2.59 days in the HFNC group, respectively. The length of ICU stay showed a significant difference (*p* < 0.01). The total length of hospitalization in the COT group was 15.50 ± 4.06 days, compared with 12.89 ± 3.80 days in the HFNC group (*p* < 0.01) (Table 2).

There was no difference in mortality during hospitalization between the two groups (1/164 in the HNFC group and 2/119 in the COT group; *p* > 0.05) (Tables 2, 7).

**TABLE 7:** Logistics regression analysis of mortality rate.

	Intercept and variable	Coefficient	Odds ratio (95%CI)	*p*-value
Mortality	Itercept	10.747	46512.673	0.290
Rate	Gender	0.349	1.418 (0.058–34.937)	0.831
	Age	0.066	1.068 (0.961–1.188)	0.224
	APACHE II	–0.009	0.991 (0.682–1.441)	0.962
	GCS_admission	–2.069	0.126 (0.015–1.080)	0.059
	Diagnosis	–0.262	0.769 (0.120–4.926)	0.782
	Oxygen therapy	–2.411	0.090 (0.004–2.110)	0.135

Multiple linear regression analyses the patients’ total hospital stay and length of stay in ICU, respectively. We have found that oxygen therapy was a significant factor in both total lengths of hospitalization (*p* < 0.01) and length of stay in ICU (*p* < 0.01) (Tables 8, 9).

**TABLE 8:** Linear regression analysis on length of ICU stay.

	Intercept and variable	B	SE	β	t	*p*-value
ICU stay	Itercept	17.980	1.381		13.017	0.000
	Gender	0.247	0.299	0.037	0.826	0.409
	Age	0.001	0.012	0.003	0.050	0.960
	APACHE II	0.092	0.054	0.125	1.712	0.088
	GCS_admission	–1.063	0.113	–0.558	–9.402	0.000*
	Diagnosis	0.016	0.102	0.007	0.157	0.876
	Oxygen therapy	–2.408	0.278	–0.381	–8.668	0.000*

*<0.05 indicate the significant result in the assessment.

**TABLE 9:** Linear regression analysis on length of hospitalization.

	Intercept and variable	*B*	SE	β	*t*	*p*-value
Hospitalization	Itercept	23.105	1.840		12.559	0.000
	Gender	0.309	0.398	0.035	0.776	0.438
	Age	–0.022	0.016	–0.086	–1.428	0.154
	APACHE II	0.348	0.072	0.359	4.849	0.000*
	GCS_admission	–0.994	0.151	–0.396	–6.599	0.000*
	Diagnosis	0.496	0.136	0.169	3.648	0.000*
	Oxygen therapy	–3.412	0.370	–0.410	–9.219	0.000*

*<0.05 indicate the significant result in the assessment.

## Discussion

In neurologically critically ill patients, pulmonary complications are still a serious challenge (Ji et al., 2013). Besides a longer hospitalization duration, this could result in a higher mortality rate (Maramattom et al., 2006; Park et al., 2021). We found that patients treated with HFNC had a lower mechanical ventilation requirement, thinner sputum, shorter ICU stays, and shorter overall hospitalization. To clarify the effect of HFNC in neurologic critic illness, 283 patients were enrolled for this retrospective analysis. Our study first reported HFNC could improve respiratory function and prevent pulmonary complications in neurologically critically ill patients.

In this study, patients with HFNC treatment were inclined to avoid mechanical ventilation (Table 2). Vargas et al. (2015) reported that HFNC improved respiration in critical care subjects. HFNC could reduce intubation rates in the ICU (Maggiore et al., 2014; Frat et al., 2015; Hernandez et al., 2016). These results are consistent with our findings. Endotracheal intubation was associated with mortality in ICH patients (Lioutas et al., 2018). Regression analysis also proved HFNC is an important influencing factor (Table 3). Our research supports HFNC could be beneficial for severely neurotic patients.

Compared with the incidence of pneumonia, no significant statistical difference was found between the two groups (Table 2). Watanabe et al. (2021) reported HFNC improved swallowing function and prevented aspiration pneumonia in a patient with low cervical spinal cord injury (CSCI) compared with COT. We found that the severity of the disease (APACHE II) was also an important factor (Table 4) by multivariable logistic regression analyses. We compared the baseline of the two groups of patients and found that the APACHE II score of the COT group was better than that of the HFNC group (Table 1). So, it could have a positive effect on the prevention of pneumonia.

Patients with severe neurological diseases are often accompanied by decreased airway protection function. HFNC can provide high flow gas with heating and humidification (Spoletini et al., 2015; Nishimura, 2016, 2019). Although HFNC did not reduce the incidence of pneumonia during hospitalization, it significantly made patients’ sputum thinner, which was conducive to sputum drainage (Table 5). This may be the reason there is an inclination to prevent mechanical ventilation in the HFNC group.

**FIGURE 2:**
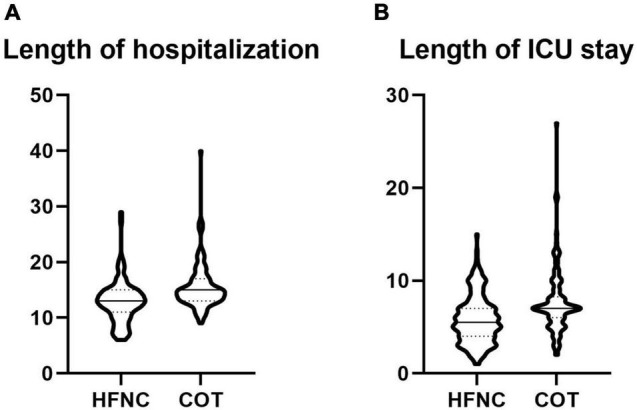
**(A)** Comparison of length of hospitalization between two groups; **(B)** comparison of length of ICU stay between two groups **(A,B)**.

Shorter ICU stays or hospital stays often mean faster recovery and less cost. Patients treated with HFNC have shorter ICU stay and overall length of hospitalization (Figure 2). A study on COVID-19 also finds that early HFNC treatment can shorten the length of hospital stay and ICU stay (Deng et al., 2021). Another study shows that HFNC combined with an external diaphragmatic pacemaker (EDP) can significantly shorten the length of NICU stay in patients with severe intracerebral hemorrhage (Yan et al., 2020). Wang et al. (2020) found the ICU stay of the HFNC group was shorter than the NIV group. Through linear regression analysis, we found that HFNC was one of the important factors for both of them (Tables 8, 9). At present, there was no study on the therapeutic effect of HFNC for severe neurological diseases. Our data supported that HFNC treatment might shorten the length of ICU stay and hospital stay in these patients.

**FIGURE 3:**
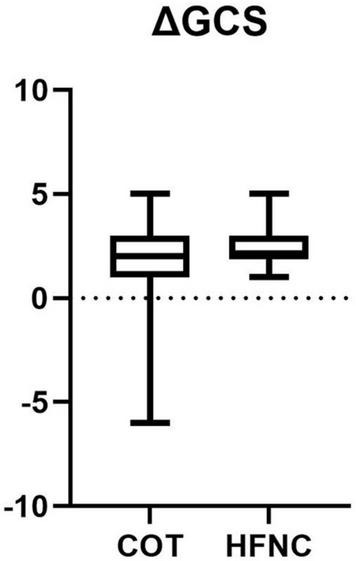
Comparison of ΔGCS between two groups.

The neurological outcome should be taken into consideration when assessing the outcome of neurological critic-illness patients. Studies showed that respiratory complications induced adverse effects on patients with severe neurological diseases (Ji et al., 2013; Divani et al., 2015; Park et al., 2021). We found that HFNC improved not only respiratory outcomes but also short-term neurological outcomes (Figure 3). We also found that factors affecting short-term neurological outcomes included age, the severity of disease (APACHE II), admission GCS score, and oxygen therapy. HFNC may improve the oxygenation of patients and reduce the secondary damage to the nervous system. Our study supports that HFNC is suitable for the treatment of elderly patients, patients with serious diseases, and patients with the neurological critical disease.

There was no difference in mortality during hospitalization between the two groups in our study. Some studies showed that HFNC reduced mortality in hospitals (Frat et al., 2015; Kim et al., 2018; Nagata et al., 2018; Deng et al., 2021), while other studies showed that HFNC did not (Wang et al., 2020). We speculated that the severity of the primary disease might be the main factor affecting mortality during hospitalization, which needs further study.

We have first reported that the role of HFNC in the treatment of severe neurological diseases, and respiratory complications in severe neurological patients. The prevention of respiratory complications can improve the short-term neurological prognosis, and shorten the ICU stay and the length of hospitalization. HFNC treatment for patients with severe neurological diseases requiring oxygen therapy may be beneficial to improve their prognosis and shorten their hospital stay.

That conclusion is based on an analysis of clinical factors. There are several limitations: first, this is a retrospective study, and further prospective studies may be needed to verify our conclusions; second, the sample size is not large enough. More patients will be included in future studies.

## Data Availability Statement

The original contributions presented in the study are included in the article/supplementary material, further inquiries can be directed to the corresponding author/s.

## Ethics Statement

The studies involving human participants were reviewed and approved by the Ethical Committee of Tianjin Medical University General Hospital Airport Hospital. The patients/participants provided their written informed consent to participate in this study.

## Author Contributions

SW carried out the statistical analyses and wrote the article. JY, YX, and HY collected the data and reviewed the article. BY, YZ, and ZW helped interpret results and reviewed the article. PZ designed and supervised the study and collected the data and edited the article. All authors contributed to the article and approved the submitted version.

## Conflict of Interest

The authors declare that the research was conducted in the absence of any commercial or financial relationships that could be construed as a potential conflict of interest.

## Publisher’s Note

All claims expressed in this article are solely those of the authors and do not necessarily represent those of their affiliated organizations, or those of the publisher, the editors and the reviewers. Any product that may be evaluated in this article, or claim that may be made by its manufacturer, is not guaranteed or endorsed by the publisher.

## References

[B1] Chanques G. Riboulet F. Molinari N. Carr J. Jung B. Prades A. (2013). Comparison of three high flow oxygen therapy delivery devices: a clinical physiological cross-over study. *Minerva Anestesiol.* 79 1344–1355. 23857440

[B2] Deng L. Lei S. Wang X. Jiang F. Lubarsky D. A. Zhang L. (2021). Course of illness and outcomes in older COVID-19 patients treated with HFNC: a retrospective analysis. *Aging* 13 15801–15814. 10.18632/aging.203224 34182540 PMC8266360

[B3] Divani A. A. Hevesi M. Pulivarthi S. Luo X. Souslian F. Suarez J. I. (2015). Predictors of nosocomial pneumonia in intracerebral hemorrhage patients: a multi-center observational study. *Neurocrit. Care* 22 234–242. 10.1007/s12028-014-0065-x 25231530

[B4] Frat J. P. Brugiere B. Ragot S. Chatellier D. Veinstein A. Goudet V. (2015). Sequential application of oxygen therapy via high-flow nasal cannula and noninvasive ventilation in acute respiratory failure: an observational pilot study. *Respir. Care* 60 170–178. 10.4187/respcare.03075 25294935

[B5] Gaddam S. S. Buell T. Robertson C. S. (2015). Systemic manifestations of traumatic brain injury. *Handb. Clin. Neurol.* 127 205–218. 10.1016/B978-0-444-52892-6.00014-3 25702219

[B6] Hernandez G. Vaquero C. Colinas L. Cuena R. Gonzalez P. Canabal A. (2016). Effect of postextubation high-flow nasal cannula vs noninvasive ventilation on reintubation and postextubation respiratory failure in high-risk patients: a randomized clinical trial. *JAMA* 316 1565–1574. 10.1001/jama.2016.14194 27706464

[B7] Hu P. J. Pittet J. F. Kerby J. D. Bosarge P. L. Wagener B. M. (2017). Acute brain trauma, lung injury, and pneumonia: more than just altered mental status and decreased airway protection. *Am. J. Physiol. Lung cell. Mol. Physiol.* 313 L1–L15. 10.1152/ajplung.00485.2016 28408366

[B8] Ji R. Wang D. Shen H. Pan Y. Liu G. Wang P. (2013). Interrelationship among common medical complications after acute stroke: pneumonia plays an important role. *Stroke* 44 3436–3444. 10.1161/STROKEAHA.113.001931 24178914

[B9] Kim E. S. Lee H. Kim S. J. Park J. Lee Y. J. Park J. S. (2018). Effectiveness of high-flow nasal cannula oxygen therapy for acute respiratory failure with hypercapnia. *J. Thorac. Dis.* 10 882–888. 10.21037/jtd.2018.01.125 29607161 PMC5864634

[B10] Lioutas V. A. Marchina S. Caplan L. R. Selim M. Tarsia J. Catanese L. (2018). Endotracheal intubation and in-hospital mortality after intracerebral hemorrhage. *Cerebrovasc. Dis.* 45 270–278. 10.1159/000489273 29898436

[B11] Maggiore S. M. Idone F. A. Vaschetto R. Festa R. Cataldo A. Antonicelli F. (2014). Nasal high-flow versus venturi mask oxygen therapy after extubation. Effects on oxygenation, comfort, and clinical outcome. *Am. J. Respir. Criti. Care Med.* 190 282–288. 10.1164/rccm.201402-0364OC 25003980

[B12] Maramattom B. V. Weigand S. Reinalda M. Wijdicks E. F. Manno E. M. (2006). Pulmonary complications after intracerebral hemorrhage. *Neurocrit. Care* 5 115–119. 10.1385/NCC:5:2:115 17099257

[B13] Nagata K. Kikuchi T. Horie T. Shiraki A. Kitajima T. Kadowaki T. (2018). Domiciliary high-flow nasal cannula oxygen therapy for patients with stable hypercapnic chronic obstructive pulmonary disease. A multicenter randomized crossover trial. *Ann. Am. Thorac. Soc.* 15 432–439. 10.1513/AnnalsATS.201706-425OC 29283682

[B14] Nishimura M. (2016). High-Flow nasal cannula oxygen therapy in adults: physiological benefits, indication, clinical benefits, and adverse effects. *Respir. Care* 61 529–541. 10.4187/respcare.04577 27016353

[B15] Nishimura M. (2019). High-Flow nasal cannula oxygen therapy devices. *Respir. Care* 64 735–742. 10.4187/respcare.06718 31110041

[B16] Park C. Charalambous L. T. Yang Z. Adil S. M. Hodges S. E. Lee H. J. (2021). Inpatient mortality and healthcare resource utilization of nontraumatic intracerebral hemorrhage complications in the US. *J. Neurosurg.* 10.3171/2020.8.JNS201839 33482635

[B17] Rittayamai N. Tscheikuna J. Rujiwit P. (2014). High-flow nasal cannula versus conventional oxygen therapy after endotracheal extubation: a randomized crossover physiologic study. *Respir. Care* 59 485–490. 10.4187/respcare.02397 24046462

[B18] Spoletini G. Alotaibi M. Blasi F. Hill N. S. (2015). Heated humidified high-flow nasal oxygen in adults: mechanisms of action and clinical implications. *Chest* 148 253–261. 10.1378/chest.14-2871 25742321

[B19] Stueber T. Karsten J. Voigt N. Wilhelmi M. (2017). Influence of intraoperative positive end-expiratory pressure level on pulmonary complications in emergency major trauma surgery. *Arch. Med. Sci.* 13 396–403. 10.5114/aoms.2016.59868 28261294 PMC5332443

[B20] Vargas F. Saintleger M. Boyer A. Bui N. H. Hilbert G. (2015). Physiologic effects of high-flow nasal cannula oxygen in critical care subjects. *Respir. Care* 60:1369. 10.4187/respcare.03814 25944940

[B21] Wang Y. Ni Y. Sun J. Liang Z. (2020). Use of high-flow nasal cannula for immunocompromise and acute respiratory failure: a systematic review and meta-analysis. *J. Emerg. Med.* 58 413–423. 10.1016/j.jemermed.2020.01.016 32220545

[B22] Watanabe Y. Tamura T. Imai R. Maruyama K. Iizuka M. Ohashi S. (2021). High-flow nasal cannula oxygen therapy was effective for dysphagia associated with respiratory muscle paralysis due to cervical spinal cord injury: a case report. *Medicine (Baltimore)* 100:e26907. 10.1097/MD.0000000000026907 34397924 PMC8360423

[B23] Yan B. Chen F. Liu Y. F. Zhao G. F. Zhang Y. S. Yin S. M. (2020). Application of external diaphragm pacemaker combination with high-flow nasal cannula in offline patients with postoperative severe cerebral hemorrhage. *Zhonghua yi xue za zhi* 100 1091–1094. 10.3760/cma.j.cn112137-20200217-00307 32294874

